# Rethinking rehabilitation resources: an amuse-bouche to supply chain management

**DOI:** 10.3389/fresc.2026.1786233

**Published:** 2026-03-18

**Authors:** Yousef Abdulsalam, Ahmad J. Abdulsalam, Levent Özçakar

**Affiliations:** 1 Department of Information Systems and Operations Management, College of Business Administration, Kuwait University, Kuwait City, Kuwait; 2 Department of Physical Medicine and Rehabilitation, Mubarak Alkabeer Hospital, Jabriya, Kuwait; 3 Department of Physical and Rehabilitation Medicine, Hacettepe University Medical School, Ankara, Türkiye

**Keywords:** healthcare resilience, Purchasing Portfolio, rehabilitation medicine, service continuity, supply chain management

## Abstract

Supply chain disruptions can compromise rehabilitation service delivery, yet this intersection remains largely unexplored in rehabilitation medicine literature. Despite growing recognition that supply chain resilience is a determinant of healthcare quality, no prior work has systematically applied supply chain frameworks to the rehabilitation context. This perspective addresses that gap by introducing supply chain management principles to rehabilitation practitioners. Drawing on the Purchasing Portfolio Model, we classify rehabilitation supplies into four categories based on cost and complexity: strategic items (customized prosthetics and orthotics), leverage items (mobility aids), bottleneck items (medications), and non-critical items (disposables). Each category requires distinct procurement and risk mitigation strategies. We discuss fundamental supply management approaches including value analysis teams that combine clinical and administrative expertise, strategic supplier relationship management, and collaborative inventory models such as purchasing alliances and consolidated service centers. Using the COVID-19 pandemic as a case in point, we illustrate that disruptions across all supply categories, from high-cost equipment to inexpensive disposables, can compromise patient care regardless of item cost. We argue that rehabilitation medicine requires a tailored supply chain literacy that bridges clinical and operational expertise. Future research should empirically examine the relationships between supply chain resilience and patient outcomes in rehabilitation settings.

## 1 Introduction

Supply chain management, broadly understood as the coordination of sourcing, procurement, storage, and distribution of goods and services across organizational boundaries, is an essential function in healthcare delivery. When functioning smoothly, these activities remain invisible to most stakeholders. While abundant research examines the use and efficacy of clinical supplies and technologies, far less attention is given to the supply chain processes that connect supplies to clinical care (1). Even mild supply disruptions can compromise patient care, while severe disruptions can escalate to healthcare delivery crises. The COVID-19 pandemic brought this reality into sharp focus, affecting all health specialties not only through its biological toll but also through constrained supply chains and widespread shortages. The U.S. Department of Health and Human Services reported that 80 percent of hospital facilities were negatively impacted at the pandemic's height, regardless of specialty, size, or location (2).

As physiatrists, we increasingly face challenges that extend beyond clinic and hospital walls. In the context of this paper, we define rehabilitation supplies broadly to encompass the physical goods required for rehabilitation service delivery: therapeutic equipment (e.g., parallel bars, ultrasound units), assistive devices (e.g., prosthetics, orthotics, wheelchairs), pharmaceuticals (e.g., analgesics, antispasmodics), and consumables (e.g., disposable gloves, electrodes). Although rehabilitation centers are generally considered less supply-dependent than surgical or cardiac facilities, supplies constitute the second largest expense category after salaries in most hospitals (3). Our patients’ recovery paths require access to equipment, assistive devices, and medications that move through complex supply networks subject to considerable risks and uncertainties (4). Even seemingly inconsequential supplies such as personal protective equipment, when disrupted, can severely impair service delivery at sophisticated hospitals (5). When essential equipment is in short supply, therapy options narrow. When medications are unavailable, clinical decision-making is constrained. Delays in assistive devices prolong hospital stays and postpone functional recovery.

Understanding how sound rehabilitation science is complemented by a resilient supply chain is therefore central to building more robust healthcare systems. This perspective argues that rehabilitation medicine, despite being perceived as a low supply-intensity specialty, is meaningfully vulnerable to supply chain disruptions and that established supply chain frameworks can provide rehabilitation professionals with actionable strategies for mitigating these risks. Specifically, we (a) apply the Purchasing Portfolio Model (6) to classify and conceptualize rehabilitation supplies according to cost and complexity, (b) introduce three fundamental supply chain strategies (value analysis, supplier relationship management, and collaborative inventory models) and assess their applicability to rehabilitation settings, and (c) identify knowledge gaps and propose future research directions for improving supply chain resilience in rehabilitation medicine.

As a perspective article, this work adopts a conceptual and narrative approach rather than a systematic review methodology. We draw on established supply chain management theory, particularly the Purchasing Portfolio Model (6) and the Sourcing Continuum framework (7), and apply these to rehabilitation medicine through illustrative examples from published literature, government reports, and documented supply disruption events. The examples cited were selected to represent each quadrant of the Purchasing Portfolio Model and to illustrate diverse disruption scenarios across supply categories. We acknowledge that this approach does not constitute empirical validation, and the framework's applicability to rehabilitation-specific contexts requires future empirical testing.

## 2 Supply categories and their implications

**Figure 1:**
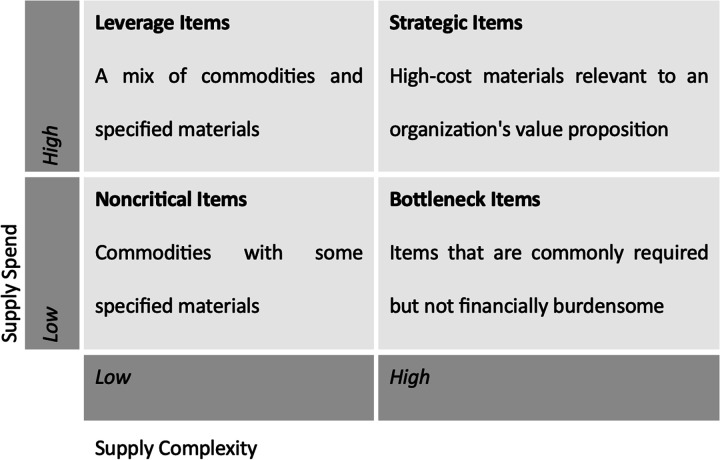
The purchasing portfolio model classifying rehabilitation supplies based on supply spend (cost × volume) and supply complexity (customization, criticality, manufacturing complexity). Adapted from Kraljic (6).

Therapists are often compelled to change, postpone, or cancel treatment plans when familiar tools and supplies are unavailable, which may affect patients’ clinical progress and potentially prolong disability. However, not all supply disruptions are alike: they vary in their causes, business impact, clinical consequences, and optimal response. The diversity of rehabilitation supplies therefore calls for a structured classification framework. The Purchasing Portfolio Model, introduced by Kraljic (6) and widely adopted in manufacturing and healthcare procurement (8), provides such a framework by classifying items along two dimensions: supply spend and supply complexity (Figure 1). Although the model was not originally designed for healthcare, it has been successfully adapted to hospital supply management (7) and offers a useful lens for rehabilitation-specific analysis.

This two-by-two matrix classifies supplies based on two dimensions: supply spend (the product of unit cost and purchasing volume) and supply complexity (encompassing degree of customization, clinical criticality, risk to patient health, and the specialized knowledge required for correct use). It is worth noting that these dimensions are context-dependent; an item classified as strategic in one rehabilitation setting may be categorized differently in another based on patient population, case mix, and institutional capacity. Four categories emerge from these dimensions, each carrying distinct implications for procurement strategy and risk management.

### 2.1 Strategic items

Customized prosthetics and orthotics exemplify strategic items, exhibiting high costs and complexity. These are characterized by complex manufacturing, limited suppliers, and are considered mission-critical supplies heavily reflecting on service strategy.

### 2.2 Leverage items

Mobility aids and exercise equipment, such as wheelchairs and exercise bikes, are expensive but relatively low complexity. This allows substitutability in product choice and flexibility in brand selection without significantly changing the experience for clinicians or patients.

### 2.3 Bottleneck items

Many drugs, such as pain medications and injectables, are examples of bottleneck items: relatively inexpensive items that are mission-critical to patient recovery and pose serious risk to patient safety if unavailable or incorrectly administered.

### 2.4 Non-critical items

General commodities used in administering care and providing patient comfort, including bed linens, office supplies, and disposable gloves.

These supply categories diverge considerably in their risk profiles and require different considerations when sourcing, procuring, storing, and dispensing items. While all categories share the risk of supply disruptions, continuity strategies should differ based on the nature of the items involved. For instance, the shortage of basic equipment like parallel bars and functional electrical stimulation devices (leverage items) forces clinicians to improvise with suboptimal alternatives (9). In contrast, disruptions in strategic items such as robotic-assisted therapy devices may alter treatment trajectories and immobilize healthcare services.

Disruption risk tends to compound with complex supplies because of their numerous subcomponents and longer manufacturing chains. For example, the global semiconductor shortage (2020–2023) affected rehabilitation equipment requiring microchips, including computerized gait training systems, functional electrical stimulation units, and high-tech prosthetics (10). Rehabilitation facilities subsequently experienced delays in acquiring new devices, and maintaining existing equipment became more difficult (11). Pediatric prosthetics, which require high customization and depend on a limited pool of qualified suppliers, illustrate how vulnerable young patients are to such disruptions (11). It should be noted, however, that the severity of these disruptions likely varies by geographic region, institutional purchasing power, and national procurement infrastructure.

Managers may intuitively prioritize continuity of costly items and overlook low-cost items, but disruptions in the latter can produce equally significant clinical consequences. When common drugs like baclofen run low, managing muscle spasticity in stroke or spinal cord injury patients becomes considerably more difficult (12). Delays in botulinum toxin can disrupt carefully timed treatment plans. Even routine analgesics, when unavailable, can hinder patients’ participation in rehabilitation. The COVID-19 pandemic's face mask shortage illustrates this phenomenon: a non-critical, low-cost consumable item, when unavailable, left physicians unable to perform their roles safely (13). This observation aligns with the supply chain literature, which emphasizes that supply criticality does not always correlate with cost (14).

## 3 Fundamentals of a resilient supply strategy

Addressing the supply chain vulnerabilities described above requires deliberate strategies for how rehabilitation supplies are sourced, delivered, and managed. Facilities must balance competing objectives: cost containment, service level maintenance, and clinical quality. The supply chain management literature identifies no universally optimal strategy; rather, effective approaches depend on institutional context, supply category, and stakeholder involvement (7). We highlight three fundamental aspects of supply strategy that are particularly relevant to rehabilitation settings, drawing on evidence from general healthcare supply chain research while noting where rehabilitation-specific validation is needed.

### 3.1 What supplies to buy?

Traditionally, physicians serve as surrogate buyers, selecting medical supplies based on their training, experience, and contact with suppliers who provide technical support (15). However, physicians naturally focus on clinical aspects, potentially overlooking cost, availability, and serviceability. A survey in orthopedics found that although physicians consider cost “very important,” only 21 percent could correctly estimate (within 25 percent margin) the costs of products they select (16). Furthermore, relationships between vendors and physicians can skew incentives (17).

Value analysis teams mitigate these issues. These committees consisting of physician leaders, procurement professionals, and biomedical engineers study supply alternatives and select a limited set of items. Physician leaders influence peers to use standardized supplies, while procurement professionals establish stronger supplier relationships. Value analysis teams can also disseminate innovative practices; tools like virtual reality systems, smartphone therapy apps, and telerehabilitation can maintain patient engagement when traditional equipment is unavailable (18).

### 3.2 Who to buy from?

Sometimes more important than selecting products is selecting suppliers. Should hospitals source directly from manufacturers for strategic partnerships and better rates, or through distributors offering multiple products per delivery? Key sourcing decisions (single vs. dual sourcing, direct vs. intermediary, local vs. global) remain active research topics in operations management (19). Partnering with multiple vendors hedges risks, while single-vendor sourcing can improve collaboration potential and pricing. Important in supplier selection is ensuring fit with the healthcare system's business strategy (20).

**Figure 2:**
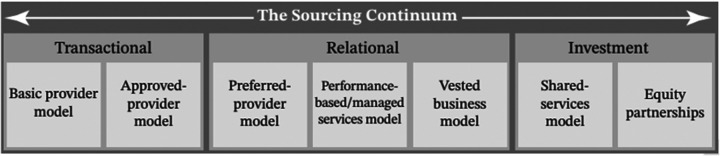
The sourcing Continuum illustrating the spectrum of buyer-supplier relationships from transactional to strategic partnerships. Adapted from Schneller et al. (7).

Strategic partners can provide healthcare systems with access to latest medical technologies, high degrees of product customization, and collaboration opportunities for research (21). For leverage and commodity items, periodically scanning the market for better prices provides cost-efficiency. Between these extremes exists a spectrum of buyer-supplier relationship modes, as demonstrated by the Sourcing Continuum model (Figure 2) (7).

### 3.3 How much, how often, and when to buy?

Forecasting is essential, incorporating delivery lead times, desired service levels, and expected patient demand. Health systems must balance ordering large batches for storage against smaller, frequent orders on an as-needed basis. Lean inventory management has gained wide adoption in healthcare, with facilities ordering only as needed and maintaining minimal stock levels for agility and efficiency (22). However, a major limitation of lean management is its reduced resiliency: supply chains that carry minimal inventory lack buffers to absorb unexpected disruptions (23). The COVID-19 pandemic exposed this trade-off, as facilities that had embraced lean practices found themselves acutely vulnerable to sudden demand surges and upstream supply failures (4).

While rival health systems may compete clinically, they need not compete logistically. Inter-system collaboration often provides net benefits for all parties. Pooling inventory provides economies of scale and diversifies demand risk through the square root law (24). Hospitals commonly form purchasing alliances to negotiate large contracts collectively, a dominant purchasing model in the U.S. that accounts for a substantial share of hospital supply spending (25). Consolidated Service Centers represent another collaborative model in which hospital consortiums establish shared service organizations, reducing duplication in personnel and infrastructure (26). Finally, value analysis teams should consider reverse logistics, including the support, maintenance, repair, and disposal services provided by suppliers, which are often overlooked in procurement decisions.

## 4 Discussion

The intersection between supply chain management and rehabilitation care has received little scholarly attention, yet it carries significant implications for clinical practice, operational planning, and health policy. Supply chain management, defined formally as the systemic coordination of key business functions across organizational boundaries (27), shapes the availability and quality of clinical resources. This perspective contributes to the literature in three ways. First, it translates the Purchasing Portfolio Model (6) into the rehabilitation context, providing a structured taxonomy for classifying rehabilitation supplies by cost and complexity. Second, it introduces rehabilitation clinicians to evidence-based supply chain strategies that have demonstrated effectiveness in broader healthcare settings but remain untested in rehabilitation. Third, it identifies a research agenda at the interface of supply chain science and rehabilitation medicine.

The classification framework presented here enables clinicians and administrators to anticipate disruption risks that differ by supply category and to develop targeted contingency plans accordingly. Importantly, as our analysis illustrates, disruptions are not confined to high-cost or high-complexity items. Low-cost consumables and routine medications can produce equally significant clinical consequences when unavailable, a finding consistent with broader supply chain research emphasizing that criticality and cost are not always aligned (14). Evidence from hospital purchasing alliances further suggests that collaborative procurement structures can mitigate such risks across supply categories (28). One important counterargument to our framework is that the Purchasing Portfolio Model, originally designed for manufacturing procurement, may oversimplify the clinical complexity of healthcare supply decisions, where patient safety considerations can override cost-based categorizations. We acknowledge this limitation and suggest that the model be used as a starting point for structured thinking rather than as a rigid classification scheme.

The three strategies discussed (value analysis teams, strategic supplier relationships, and collaborative inventory models) have demonstrated effectiveness in surgical and acute care settings (7, 25, 26), but their specific application to rehabilitation medicine remains empirically untested. Several concrete research questions emerge from this analysis. First, how do supply chain disruptions in rehabilitation settings affect measurable patient outcomes such as functional independence scores, length of stay, and readmission rates? Second, what is the cost-effectiveness of different inventory strategies (lean vs. buffered) for rehabilitation-specific supply categories? Third, how can supply chain resilience metrics, such as those proposed in the operations management literature (29), be adapted for rehabilitation facilities? Fourth, to what extent do supply chain disruptions differentially affect rehabilitation outcomes across different healthcare systems (e.g., public vs. private, resource-rich vs. resource-constrained settings)? Addressing these questions will require collaboration between rehabilitation researchers, health services researchers, and supply chain management scholars.

Several limitations should be acknowledged. As a perspective article, this work provides a conceptual introduction rather than a systematic synthesis of evidence or empirical analysis. The Purchasing Portfolio Model and Sourcing Continuum framework, while well-established in the supply chain literature, were not developed for healthcare and have not been empirically validated within rehabilitation-specific contexts. The examples and illustrations presented are drawn primarily from published reports and documented disruption events, largely from the United States, and may not generalize to all healthcare systems or geographic regions. Healthcare supply chain dynamics vary considerably across national health systems, and strategies that are effective in one institutional or regulatory context may require substantial adaptation elsewhere. Furthermore, we have focused on physical goods (equipment, devices, pharmaceuticals, consumables) and have not addressed workforce supply, facility capacity, or information systems, all of which also constitute critical inputs to rehabilitation service delivery. Finally, the perspective does not incorporate the viewpoints of patients, who are the ultimate stakeholders affected by supply disruptions, and future work should consider patient-reported outcomes and experiences.

This perspective makes the case that rehabilitation medicine, while often perceived as a low supply-intensity specialty, is in fact meaningfully vulnerable to supply chain disruptions that can directly affect patient care and clinical outcomes. By applying the Purchasing Portfolio Model to rehabilitation supplies and introducing evidence-based supply strategies from the broader healthcare literature, we offer rehabilitation professionals a structured entry point into supply chain thinking. For clinical practice, the framework encourages proactive engagement between clinicians and procurement teams, with the goal of developing category-specific contingency plans before disruptions occur. For health policy, our analysis supports the inclusion of rehabilitation supply needs in national stockpile planning and pandemic preparedness frameworks. For research, we have identified a set of empirically testable questions at the intersection of supply chain science and rehabilitation medicine. Ultimately, building supply chain literacy among rehabilitation professionals and fostering interdisciplinary collaboration between clinicians, administrators, and supply chain scholars will be essential for maintaining high-quality patient care in an increasingly uncertain global environment.

## Data Availability

The original contributions presented in the study are included in the article/Supplementary Material, further inquiries can be directed to the corresponding author.
