# Correction: Rethinking rehabilitation resources: an amuse-bouche to supply chain management

**DOI:** 10.3389/fresc.2026.1864524

**Published:** 2026-05-27

**Authors:** Yousef Abdulsalam, Ahmad J. Abdulsalam, Levent Özçakar

**Affiliations:** 1 Department of Information Systems and Operations Management, College of Business Administration, Kuwait University, Kuwait City, Kuwait; 2 Department of Physical Medicine and Rehabilitation, Mubarak Alkabeer Hospital, Jabriya, Kuwait; 3 Department of Physical and Rehabilitation Medicine, Hacettepe University Medical School, Ankara, Türkiye

**Keywords:** healthcare resilience, purchasing portfolio, rehabilitation medicine, service continuity, supply chain management

The reference for 20 was erroneously written as “Yildiz H, Babich V, Beil DR, Duenyas I, Rajan B. When complexity meets complexity: cOVID-19-induced supply chain disruptions and strategy portfolio efficiency. J Oper Manag. (2025) 71:109–29. doi: 10.1002/joom.1299”. It should be “Yildiz H, Yan T, Hatton M, Fowler J, Kull TJ, Sisk L. When complexity meets complexity: COVID-19-induced supply chain disruptions and strategy portfolio efficiency. J Oper Manag. (2025) 71:109–29. doi: 10.1002/joom.1347”

The original version of this article has been updated.

